# Stimuli-Responsive Hydrogels for Protein Delivery

**DOI:** 10.3390/gels9100802

**Published:** 2023-10-06

**Authors:** Rafaela Malta, Ana Camila Marques, Paulo Cardoso da Costa, Maria Helena Amaral

**Affiliations:** 1 CeNTI—Centre for Nanotechnology and Smart Materials, Rua Fernando Mesquita, 2785, 4760-034 Vila Nova de Famalicão, Portugal; rafaelafmalta@gmail.com; 2 UCIBIO—Applied Molecular Biosciences Unit, MEDTECH, Laboratory of Pharmaceutical Technology, Department of Drug Sciences, Faculty of Pharmacy, University of Porto, R. Jorge Viterbo Ferreira 228, 4050-313 Porto, Portugal; pccosta@ff.up.pt; 3 Associate Laboratory i4HB, Institute for Health and Bioeconomy, Faculty of Pharmacy, University of Porto, R. Jorge Viterbo Ferreira 228, 4050-313 Porto, Portugal

**Keywords:** stimuli-responsive hydrogels, proteins, peptides, protein delivery

## Abstract

Proteins and peptides are potential therapeutic agents, but their physiochemical properties make their use as drug substances challenging. Hydrogels are hydrophilic polymeric networks that can swell and retain high amounts of water or biological fluids without being dissolved. Due to their biocompatibility, their porous structure, which enables the transport of various peptides and proteins, and their protective effect against degradation, hydrogels have gained prominence as ideal carriers for these molecules’ delivery. Particularly, stimuli-responsive hydrogels exhibit physicochemical transitions in response to subtle modifications in the surrounding environment, leading to the controlled release of entrapped proteins or peptides. This review is focused on the application of these hydrogels in protein and peptide delivery, including a brief overview of therapeutic proteins and types of stimuli-responsive polymers.

## 1. Introduction

Peptides and proteins perform vital functions in the human body during almost all biochemical processes, having received growing attention as drug candidates in recent years [1,2]. However, their physicochemical properties render them difficult to use as drug substances. Particularly, peptides and proteins are not ideal for oral administration, mostly because they lack stability in the gastrointestinal tract (GIT), and their hydrophilicity and size result in poor oral bioavailability [3,4,5]. There are also some disadvantages associated with other routes of administration, including intravenous injection, which may not be enough to achieve optimal therapeutic effects since various peptides and proteins have a short half-life [3,6,7]. Accordingly, significant effort has been devoted to developing drug delivery systems that allow peptides and proteins to reach their target sites more effectively.

Hydrogels have enduring popularity in protein delivery due to their suitable features, such as biocompatibility, porous structure, which enables the transport of various peptides and proteins, and protective effect against degradation [8,9]. Many studies have recently focused on stimuli-responsive hydrogels, which can modify their physicochemical characteristics in response to external stimuli (temperature, pH, enzymes, among others) [10].

In this review, a summary overview of therapeutic proteins and their delivery organized by route of administration is provided. Also, different types of stimuli-responsive hydrogels, and their application as peptide and protein delivery systems are presented.

## 2. Therapeutic Proteins

### 2.1. Characteristics

Peptides and proteins are essential biological macromolecules that have a central role inside cells during enzyme catalysis, transportation, signal transduction, gene regulation, and immunity-related functions [11]. These compounds are also involved in several pathological conditions, including cancer, diabetes, and hypertension. Therefore, considering their diversity of functions and participation in the control of various diseases, proteins and peptides are promising therapeutic agents [12,13].

Since the approval of the first protein used as an active substance—human recombinant insulin, Humulin^®^—in 1982 by the U.S. Food and Drug Administration (FDA), several therapeutic proteins have been approved for clinical usage, and others are in the process of development [1,2].

In 2019, the FDA approved 48 novel drugs, of which approximately 21% are proteins [14,15]. One year later, proteins accounted for about 25% of the 53 FDA-approved drugs [16]. The authorization of proteins in 2022 increased slightly compared to 2021 (five vs. four, respectively) [17]. Common therapeutic proteins include the fastest growing class of monoclonal antibodies, enzymes, hormones, growth factors, anticoagulants, and fragment crystallizable (Fc) fusion proteins, among others [18,19].

Therapeutic proteins can be used as drugs to (i) substitute a protein that is abnormal or deficient, (ii) increase an existing pathway, (iii) provide a new function or activity, and (iv) interfere with a molecule or organism [20].

Peptides and proteins consist of amino acid units joined together by peptide bonds. Whereas peptides contain two to fifty amino acids, macromolecules with more than fifty amino acids are known as proteins. The sequence of amino acids in their structure is designated as the primary structure [21]. Following the interaction and folding of amino acid chains, higher levels of organization arise, namely secondary, tertiary, and quaternary structures [22]. The functional characteristics of proteins rely on their three-dimensional (3D) conformation. As the 3D structure depends on the primary structure, any difference in the latter may produce a protein that is unable to perform its function [11].

**Figure 1:**
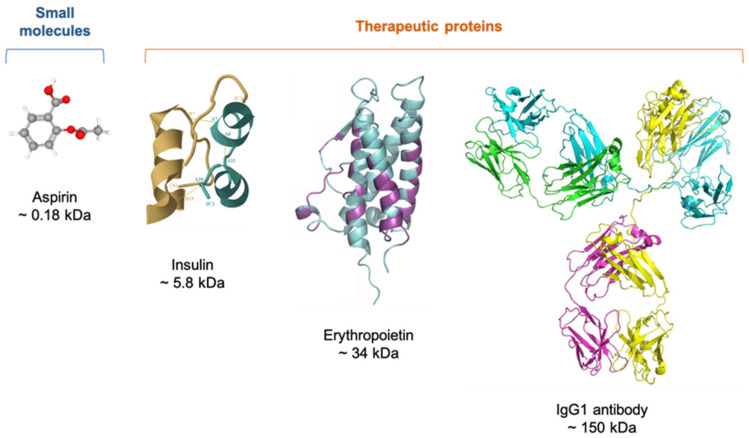
Comparison between the complexity of small molecules and therapeutic proteins.

Therapeutic proteins include molecules ranging in size from 1 to 50 kDa to much larger proteins like monoclonal antibodies (mAbs) with around 150 kDa; thus, even the smallest of these molecules exceed in size the so-called conventional drugs, such as aspirin (Figure 1) [23,24,25].

The higher molecular weight of peptides and proteins impedes them from crossing the intestine mucosa [26] and other membranes. In addition, most proteins and peptides are hydrophilic and may have groups with charges that further reduce their translocation ability through the cell membrane and are absorbed by the systemic circulation. The lipophilic nature of these membranes thus hampers the passive diffusion of relatively high hydrophilic molecules [27].

In different body regions, such as the small intestine and stomach, peptide bonds are very prone to enzymatic hydrolysis. Consequently, as therapeutic peptides and proteins may have a short circulation half-life, it is likely that biological activity is not preserved until the therapeutic effect is achieved [13].

Generally, the lower the molecular weight of a peptide or protein, the higher the metabolism and, in turn, the shorter the half-life. Likewise, proteins or peptides with higher molecular weight are related to minor metabolism and longer half-lives [5]. Proteins and peptides are sensitive to environmental changes, such as pH. By disrupting structural, noncovalent interactions, these changes can alter the native 3D structure of proteins and peptides, with loss or change in the biological activity being the outcome [13]. Ultimately, extreme pH values cause protein denaturation (unfold), rendering them inactive [4].

Due to the physicochemical properties described above, while therapeutic proteins have poor bioavailability via the oral route (less than 1–2%), parenteral administration of liquid formulations is considered the most suitable for protein delivery [3,7,28,29]. Still, the high frequency of injections reduces patient compliance on account of pain and skin wounds [6]. Therefore, together with the parenteral administration of proteins, other delivery routes, such as oral, ocular, pulmonary, nasal, and transdermal, have been explored [3,6,7]. The benefits and drawbacks of each route are described in the following sections.

### 2.2. Delivery of Therapeutic Proteins

#### 2.2.1. Parenteral Route

Therapeutic peptides and proteins are mostly administered by intravenous (IV), subcutaneous (SC), and intramuscular (IM) routes [30,31]. Although medicines can be given intravenously as a bolus, proteins are frequently administered as an infusion [32]. With the IV route, it is possible to achieve an immediate physiological response due to the complete delivery of the administrated proteins to the systemic circulation, avoiding the first-pass metabolism [33]. Notwithstanding its high bioavailability, IV administration is invasive and often painful. Moreover, treatment with high doses, as in the case of antibodies, requires infusion and, thus, visits to the hospital, which increases the overall cost of intravenously given drugs [32]. Furthermore, sterility is a critical parameter of the IV injection that also raises manufacturing costs, and some steps in the sterilization process can even affect protein stability [34].

For some polypeptides and proteins, SC administration poses an alternative to the IV route while also bypassing the first-pass metabolism. Furthermore, as the SC approach could allow patients to self-administer proteins [32], patient preference and adherence are improved, resulting in overall cost savings. Regardless of the benefits of SC-administrated proteins, it still represents an invasive route and demands patients know how to take their medication safely. Additionally, SC injection is restricted to the maximum volume of 2.0 mL because higher volumes would cause rapid changes in the hydrostatic pressure that are perceived as painful [35]. Although such a volume is usually adequate for administering peptides due to their potency, high concentrations are often necessary if proteins are the case. For instance, some antibody solutions at higher concentrations exhibit high viscosity, which might increase injection time and discomfort at the site of injection, with a negative impact on patient compliance [36]. Compared to IV administration, drugs injected subcutaneously have lower bioavailability, presumably due to catabolism at the injection site [32,37]. Also, these proteins, particularly the larger ones (>16 kDa), can show higher immunogenicity, as they preferentially drain into the lymphatic system before entering the systemic circulation [38,39].

Table 1 shows a few examples of protein-based parenteral dosage forms recently approved by the FDA [40,41,42,43,44,45,46].

#### 2.2.2. Oral Route

The preference for the oral route for drug delivery can be attributed to its ease of administration and noninvasive nature [47]. Nevertheless, delivering peptides and proteins by the oral route is very challenging.

As mentioned earlier, the low oral bioavailability of therapeutic proteins comes mainly from presystemic enzymatic degradation and limited penetration through the gastrointestinal epithelium, hence the restricted access to the systemic circulation.

The GIT contains large quantities of several enzymes, such as pepsin, trypsin, and chymotrypsin, and bile salts, which may elicit premature leakage and degradation of therapeutic proteins [48]. Moreover, the pH values in the GIT vary considerably from highly acidic (pH 2.0–4.0) in the stomach to pH ~5.5 in the duodenum, ~6.0 in the jejunum, 7.2–8.0 in the ileum, and ~6.5 in the colon, also adding difficulty for oral delivery [49].

Besides lubricating and protecting the cell layer, the thick mucus layer covering the intestinal epithelium acts as a physical barrier to the absorption of drugs, hindering contact with epithelial cells and, thus, drug transport [50]. Molecules can be electrostatically trapped in mucus by virtue of its mucin proteins and proteolytic enzymes in abundance [51]. In addition to the mucus layer, the intestinal epithelium represents a second physical barrier, consisting of a continuous monolayer of epithelial cells, such as enterocytes, goblet cells, Paneth cells, and microfold cells [48,51]. This cellular barrier regulates the transport of nutrients and proteins across the gut lumen and the bloodstream or lymphatic system [51]. The permeation of proteins and peptides between adjacent intestinal cells, designated paracellular transport, is prevented by tight junctions, having an estimated average pore radius of 8–13 Å [51,52]. For that reason, molecules larger than 0.5 kDa are not small enough to freely pass through these pores [53]. Transcellular transport, meaning the transport through epithelial cells, is normally restricted to very lipophilic molecules that readily cross the cellular barrier by passive diffusion [51]. In the case of large and often charged molecules, enterocytes or microfold cells can mediate active transport via transcytosis [54]. Still, even if the protein or peptide succeeded in penetrating the gastrointestinal mucosa, it would enter the liver by the hepatic portal vein, where first-pass metabolism takes place and further reduces the amount reaching the systemic circulation [51].

There are only a few commercially available therapeutic proteins for administration via oral route. One of the first peptide drugs approved by FDA for oral delivery is linaclotide (Linzess^®^), approved in 2012, which is both acid- and pepsin-resistant and used to treat patients with irritable bowel syndrome and chronic constipation. In 2017, semaglutide (Rybelsus^®^) was the first oral glucagon-like peptide-1 (GLP-1) approved for type 2 diabetes treatment [50].

#### 2.2.3. Nasal Route

In general, the nasal route is best suited for drug delivery as it is noninvasive and the nasal mucosa is easily accessible, considering that the epithelial barrier is thin, porous, and highly vascularized [1]. Since the nasal venous system provides direct access to the systemic circulation, the loss of drug by the hepatic first-pass metabolism can be prevented [55].

Like the intestinal epithelium, the nasal epithelium is the main physical obstacle to the passage of proteins and peptides due to their low membrane permeability [56]. It is noteworthy that nasal mucociliary clearance is a primary defense mechanism of the lungs, in which mucus and its foreign, potentially harmful substances are removed from the respiratory tract. Knowing that the mucus layer is renewed every 15–30 min, the contact time between the protein or peptide and the nasal epithelium is thus limited [57]. Even though the mucus layer can cause protein degradation by enzymatic activity in the nasal mucosa, it is relatively low when compared to that of the GIT [55]. It follows that the nasal bioavailability of peptides and proteins is usually between 1 and 3% [55,56].

#### 2.2.4. Pulmonary Route

In addition to noninvasiveness and hepatic first-pass metabolism avoidance, other advantages of the pulmonary route for drug delivery that merit attention and intensive research include (i) the large surface area of lungs, (ii) a very thin alveolar epithelium, and (iii) a rich vascular supply, allowing for rapid systemic absorption [1,56].

However, some factors affect the delivery efficacy of inhaled proteins and peptides, with the primary barrier for inhaled particle deposition being the highly branching structure of the lung [23]. The rate and extent of this process depend significantly on the physicochemical properties of aerosol particles, especially the diameter of a particle in airflow, referred to as aerodynamic diameter [56,58]. Whereas particles with aerodynamic diameters ranging from 1 to 5 μm are deposited in the lower respiratory tract, those with diameters greater than 10 μm are deposited in the oropharyngeal region [23]. Particles exhaled during tidal breathing are under 1 μm [59].

After their deposition in the lungs, therapeutic proteins can be removed by either mucociliary clearance or alveolar macrophage uptake via pinocytosis [23,60]. The latter is size-dependent and becomes more relevant to large proteins (≥40 kDa) owing to their slower transport and absorption across the alveolo-capillary barrier. Alveolar macrophage uptake may not have such an impact on small proteins and peptides (≤25 kDa) as they are readily absorbed from airspaces [60]. Therapeutic proteins also encounter enzymes in the lungs but undergo less degradation compared to the GIT [61]. It is established that proteins and peptides with molecular weights around 6–50 kDa have good bioavailability following inhalation [1,23,62].

#### 2.2.5. Ocular Route

It is the route of choice to deliver drugs directly to the ocular tissue [32]. Bearing in mind how accessible the front of the eye is, it comes as no surprise that topical instillation of eyedrops is often selected to treat diseases affecting the anterior segment of the eye, including the cornea, conjunctiva, aqueous humor, iris, ciliary body, and lens [63]. Nevertheless, less than 5% of a topically applied drug reaches deeper ocular tissues because reflex blinking and increased tear turnover collectively lead to poor drug retention and permeation [64,65]. The nasolacrimal duct drains the excess volume into the systemic circulation [64]. The rest of the protein or peptide faces the corneal epithelial barrier, formed by five to seven cell layers, also limiting its penetration [66]. Therefore, topical administration fails to deliver therapeutic concentrations of the drug to the posterior segment of the eye, consisting of the retina, vitreous, and choroid. An alternative to topical eye drops application is intravitreal injection [63,67], but vitreous humor turnover rapidly clears the drug. Moreover, while repeated injections are needed to ensure good therapeutic efficacy, frequent eye punctures with intravitreal injections are responsible for several side effects, including endophthalmitis, retinal detachment, hemorrhage, and poor patient tolerance [32,63,67].

#### 2.2.6. Transdermal Route

Skin delivery of proteins and peptides may be efficient since it bypasses the liver, allows for sustained-release effect, and has less proteolytic activity than other mucosal routes [68]. Sustained release may overcome the need for frequent injections if the protein or peptide has a short in vivo half-life [69]. Seeing that the primary function of the skin is to protect the body against exogenous substances, achieving the permeation of protein molecules through the skin is undoubtedly a challenge [56]. Acting as the first and principal barrier to the transdermal route, the topmost layer of the skin, designated stratum corneum, consists of keratinocytes embedded in a lipid matrix, highly organized in a “brick-and-mortar” formation [1,68]. Again, lipid content is a constraint on permeability to hydrophilic molecules, so the passive permeation of proteins and peptides through the stratum corneum is unattainable, not to mention their inherent low diffusivity due to high molecular weight [70]. Also, corneocyte replenishment is constant, providing an active mechanism for removing unabsorbed drugs from the body. Both chemical and physical enhancers can be used to make the skin more permeable. Notwithstanding that chemical enhancement techniques (e.g., solvents like ethanol and surfactants) are effective for small, lipophilic molecules, they cannot usually increase skin permeability to peptides and proteins. Alternatively, physical approaches (e.g., iontophoresis, sonophoresis, and microporation) have shown great promise, allowing peptides to cross the skin through a transient rearrangement or disruption of the stratum corneum structure [69,71,72]. Once therapeutic proteins pass through the stratum corneum, they must move through the viable epidermis, devoid of blood vessels, to finally reach the dermis, where systemic absorption occurs [56].

A summary of commercially available proteins and peptides is given in Table 2.

Given the limitations of each route of administration, significant strategies have been studied for developing drug delivery systems that allow proteins and peptides to reach their target sites more efficiently [8]. Recent attention has been directed towards delivery approaches based on stimuli-responsive smart materials, particularly hydrogels [9,77].

## 3. Hydrogels

### 3.1. Definition

Hydrogels are 3D, hydrophilic polymeric networks that can swell and retain significant amounts of water or biological fluids without being dissolved [10].

Over the last few decades, hydrogels have been widely used as tissue engineering scaffolds, wound dressings, medical adhesives, and contact lenses. Additionally, hydrogels are becoming increasingly attractive as vehicles for protein delivery due to their desirable properties. Hydrogels are similar in structure to the natural extracellular matrix and enable the physical incorporation of peptides and proteins [8,78]. The crosslinked nature of hydrogels is beneficial for transporting peptides and proteins, as it prevents large foreign molecules from interacting with the encapsulated proteins, thus promoting their retention in circulation without immune rejection. Besides, the high water content of hydrogels helps preserve the active form of proteins and decreases their vulnerability to chemical degradation [8,79]. Although it is assumed that protein release from the hydrogel network is controlled by diffusion, swelling, and/or erosion/degradation, other mechanisms of protein adsorption/desorption to the hydrogel structure can also be involved [78,80]. Protein encapsulation into micro/nanoparticles before dispersion in the hydrogel matrix may also affect their release.

The development of hydrogels based on stimuli-responsive polymers has gained momentum in recent years.

### 3.2. Stimuli-Responsive Polymers

**Figure 2:**
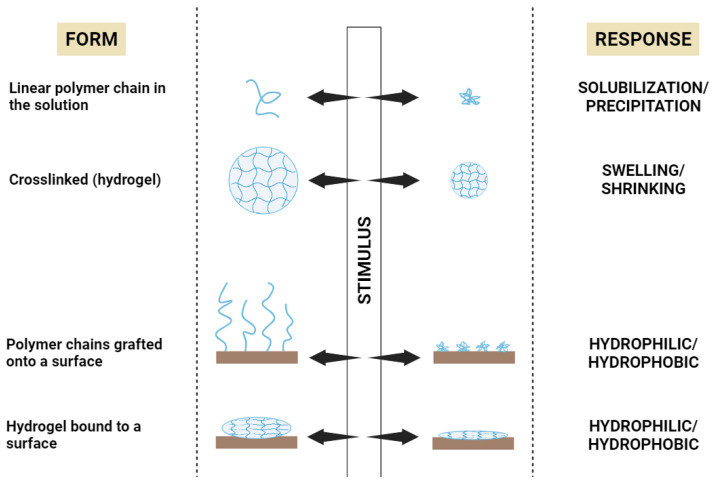
The macroscopic response of different forms of “smart” polymers [83]. Created with BioRender.com.

Stimuli-responsive hydrogels exhibit rapid physicochemical transitions in response to subtle variations in the surrounding environment, leading to the release of the entrapped molecules in a controlled manner [81]. Also termed “smart” polymers, stimuli-responsive polymers respond to external stimuli with reversible changes as they return to their original state after the stimulus is removed [82]. As illustrated in Figure 2, their macroscopic response can be a change in solubility, swelling/shrinking, or switching between hydrophilic/lipophilic, depending on whether the “smart” polymer chains are dissolved in an aqueous solvent (sol state), crosslinked forming a hydrogel, or grafted onto/bound to a surface [83].

According to their nature, stimuli might be physical (temperature, light), chemical (ionic strength, pH), or biochemical (enzyme, substrates) [84]. Stimuli can also be divided into endogenous or exogenous, depending on whether they occur naturally in the body or are artificially applied from outside the body [85].

#### 3.2.1. Temperature-Responsive Polymers

By shifting from ambient to body temperature, some temperature-responsive (or thermoresponsive) polymers undergo a sol–gel phase transition [86]. The ideal thermoresponsive polymer-based system is a free-flowing liquid at room temperature and only transforms into a gel once administered to the body [87].

Thermoresponsive polymers that form a gel with the elevation of the temperature have a lower critical solution temperature (LCST). At temperatures below the LCST, these polymers are miscible with water [88]. An upper critical solution temperature (UCST)-type behavior is identified when thermoresponsive polymers yield a gel below the critical temperature and return to the sol state above it [89]. Systems with LCST behavior in water are usually preferred for drug delivery technologies since the need for high temperatures in the UCST systems is not convenient for heat-labile biomolecules and drugs [90]. Among thermoresponsive polymers, poly(N,N-diethylacrylamide) (PDEAAm), poly(N-isopropylacrylamide) (PNIPAAm), and poloxamers (Pluronics^®^) are the most commonly used [91].

#### 3.2.2. pH-Responsive Polymers

The use of pH-responsive polymers in drug delivery systems takes into consideration that pH differences exist in the human body under normal or pathological conditions. For instance, as previously mentioned, the pH of the GIT varies greatly, with the stomach being strongly acidic and the intestine alkaline. Therefore, some pH-responsive polymers can be used to prevent gastric degradation and premature release in the stomach upon reaching the intestine [92]. pH-responsive polymers have also found applications in cancer-targeting strategies that capitalize on the acidic environment of the tumor (pH 5–6), as opposed to a normal physiological pH of 7.4 [93].

pH-responsive polymers have acidic (carboxyl) or basic (amine, imine) ionizable groups attached to the hydrophobic backbone, thus being considered polyacids (anionic) or polybases (cationic). These pendant groups can either donate or accept protons, depending on their pK_a_ and the environmental pH value [94]. Cationic hydrogels swell at a low pH (pH < pK_a_), and anionic hydrogels, on the other hand, swell at a higher pH (pH > pKa) due to the protonation of amino/imine groups and ionization of the acidic groups, respectively. As a result, electrostatic repulsion between charges leads to polymer chain expansion and impels the hydrogel to imbibe larger quantities of water [95,96]. Amino alkyl methacrylate copolymer (Eudragit E) (cationic), poly(methacrylic acid-co-methyl methacrylate) (Eudragit L/S) (anionic), and hydroxypropyl-methylcellulose phthalate (HPMC-P) (anionic) are in the forefront of pH-sensitive polymers used in drug delivery [97].

#### 3.2.3. Ionic Strength-Responsive Polymers

Gelation can occur as a response to alterations in the ionic content of the surrounding medium if ionic strength-responsive polymers are involved [98]. It is suggested that high salt concentrations reduce the repulsive electrostatic strength of the polymer, followed by an increase in hydrophobic interactions and, in turn, network precipitation [99]. Also, hydrogels made from these polymers swell differently in water and in an electrolytic solution [100]. Besides inducing hydrogelation, ionic strength is an effective way to improve mechanical and transport properties [101].

The presence of ions in physiological fluids and the mucus layer covering mucosal membranes represents a potential stimulus with particular interest for mucoadhesive and topical formulations. Moreover, a variety of medical conditions are associated with changes in ionic concentration, such as increased serum calcium levels in vascular and bone diseases or iron deficiency in anemia [102].

Compared to temperature- and pH-responsive polymers, examples of ionic-responsive polymers as smart drug delivery hydrogels are somewhat scarce. Still, it is worth mentioning that alginates can form gels in the presence of polyvalent cations, such as Ca^2+^, Mg^2+^, or Fe^2+^ [103,104]. Gellan gum also gels after being exposed to different metal ions and even hydrogen ions, although this is less noticeable [102].

#### 3.2.4. Biomolecule-Responsive Polymers

Biomolecule-responsive hydrogels can undergo structural transition in response to specific target biomolecules, such as glucose, proteins, nucleic acids, and polypeptides [105].

Glucose-responsive hydrogels can be suitable materials for diabetes management based on the glucose levels in the bloodstream. To achieve a self-regulated delivery of insulin, glucose-responsive moieties, such as glucose oxidase, lectin (concanavalin A, Con A), and phenylboronic acid (PBA), are incorporated into the hydrogel system [106].

The first approach is possible upon immobilizing glucose oxidase in a pH-responsive hydrogel enclosing a saturated insulin solution. At high glucose concentrations, glucose diffuses into the hydrogel and is oxidized to gluconic acid, prompting mesh expansion and release of previously entrapped insulin to the medium. As a result, sugar levels drop, causing a rise in pH that prevents further insulin release [107,108]. A different strategy takes advantage of the competitive binding of Con A to glucose and glycosylated insulin. Since Con A has a greater affinity for glucose, increased levels of glucose trigger the displacement and release of glycosylated insulin by diffusion across the hydrogel matrix [90]. Alternatively, PBA-based platforms can also tune insulin activity for personalized diabetes therapy. There is a dynamic equilibrium between the charged and uncharged forms of PBA in aqueous media. When the ionized form of PBA in insulin-loaded hydrogels binds to glucose, the combined effect of polymer chain repulsion and increased hydrophilicity drives insulin release along with the rapid expansion of the hydrogel [109,110].

In the case of antigen-responsive hydrogels, the ability to undergo volume or structural changes relies on antigen–antibody interactions. This group of bio-responsive hydrogels can be prepared by the (i) immobilization of antigens or antibodies within the hydrogel structure, (ii) chemical conjugation of the polymer to antigens or antibodies, and (iii) copolymerization with the antigen-binding fragment of the antibody [111]. To illustrate, grafting the polymer network with an antigen and its corresponding antibody enables a hydrogel to form upon an antigen–antibody binding. As such, free antigens found in the environment elicit a competitive binding that reduces the crosslinking density of the hydrogel and allows swelling [112,113].

Finally, hybridization between complementary DNA and RNA strands can be considered for developing DNA- and RNA-responsive hydrogels, which respond to the presence of DNA and RNA targets with volume changes and sol–gel phase transitions [105]. Another promising strategy employs single-stranded DNA or RNA molecules called aptamers, which have similar features to antibodies and great potential for molecular recognition [114].

#### 3.2.5. Enzyme-Responsive Polymers

Enzymes are increasingly used as stimuli to trigger structural transformations in hydrogels. To understand this, one should acknowledge that many medical conditions are associated with altered expression of proteins, more precisely overexpressed enzymes in diseased tissues [115].

In general, the design of enzyme-responsive hydrogels has three basic requirements. First, the hydrogel system must have substrate mimics or other elements that only enzymes can recognize [116]. For proteolytic enzymes, common recognition elements could be peptide chains/linkers or polymer–peptide conjugates with specific amino acid sequences that determine enzyme–substrate specificity [117]. A second prerequisite is the accessibility of the incorporated substrates to enzymes, otherwise the kinetics of enzyme-catalyzed reactions can be greatly affected. Lastly, enzyme–substrate reactions must be translated into changes in the hydrogel, such as morphological transformation or degradation [117,118].

Table 3 provides some examples of stimuli-responsive polymers.

#### 3.2.6. Dual and Multiple Stimuli-Responsive Polymers

On some occasions, polymer materials with a single responsiveness may not fully serve the therapeutic purpose in a complex physiological or pathological microenvironment [139]. Therefore, polymer materials that respond to various physical or chemical stimuli are in high demand for biomedical applications.

Dual stimuli-responsive polymers respond to two stimuli combined (pH/temperature, ionic strength/pH, ionic strength/temperature, temperature/enzyme, etc.). As regards multiple stimuli-responsive polymers, more than two stimuli, such as temperature/pH/redox, temperature/pH/biomolecule, or temperature/redox/biomolecule, will trigger a response [140,141].

Applying polymers with pH and temperature responsiveness is a growing trend for anticancer agents’ delivery since many tumors display elevated temperature and low pH compared to healthy tissues. The most investigated thermoresponsive polymer is pNIPAAm with an LCST of 32 °C in water; the polymer network collapses above the LCST, and the corresponding hydrogel shrinks at body temperature (37 °C). In the aforementioned context of cancer treatment, combinations of pNIPAAm and pH-responsive polymers, such as polyacrylamide and polyacrylic acid, also provide valuable options to generate dual responsiveness [142].

## 4. Stimuli-Responsive Hydrogels for Protein Delivery

Some prominent examples of stimuli-responsive hydrogels for delivering therapeutic proteins are presented below.

In a strategy to deal with the problems of protein delivery in the stomach, Lima et al. [143] chose alginate as the hydrogel matrix and bovine serum albumin (BSA) as a model protein. The resulting hydrogel showed biocompatibility and pH-dependent BSA release and swelling profile, reaching the highest value of swelling at pH 7.4. The overall results suggested that the performance of this alginate-based hydrogel as an oral drug delivery system would be excellent.

Phan et al. [144] developed an injectable hydrogel based on temperature- and pH-responsive poly(ethylene glycol)–poly(sulfamethazine carbonate urethane) copolymers for lysozyme delivery. Although lysozyme was used therein as a model protein, increasing evidence underlines its potential for clinical applications due to its antibacterial, anti-inflammatory, anticancer, and analgesic properties [145]. The obtained hydrogel showed very low cytotoxicity even at higher polymer concentrations, and further in vivo studies demonstrated a sustained release of lysozyme for seven days after SC administration in Sprague Dawley rats.

Knowing that keratinocyte growth factor (KGF) repairs potently epithelial tissue, Xu et al. [146] proposed a thermoresponsive heparin-modified poloxamer hydrogel containing KGF to prevent intrauterine adhesion, the main cause of infertility and recurrent pregnancy loss in women with reproductive capacity. In vitro studies showed a sustained release of KGF from the hydrogel. On the seventh day after injection into the intrauterine cavity, the authors observed endometrial epithelial cell growth and angiogenesis in the injured uterus of a rat model.

**Figure 3:**
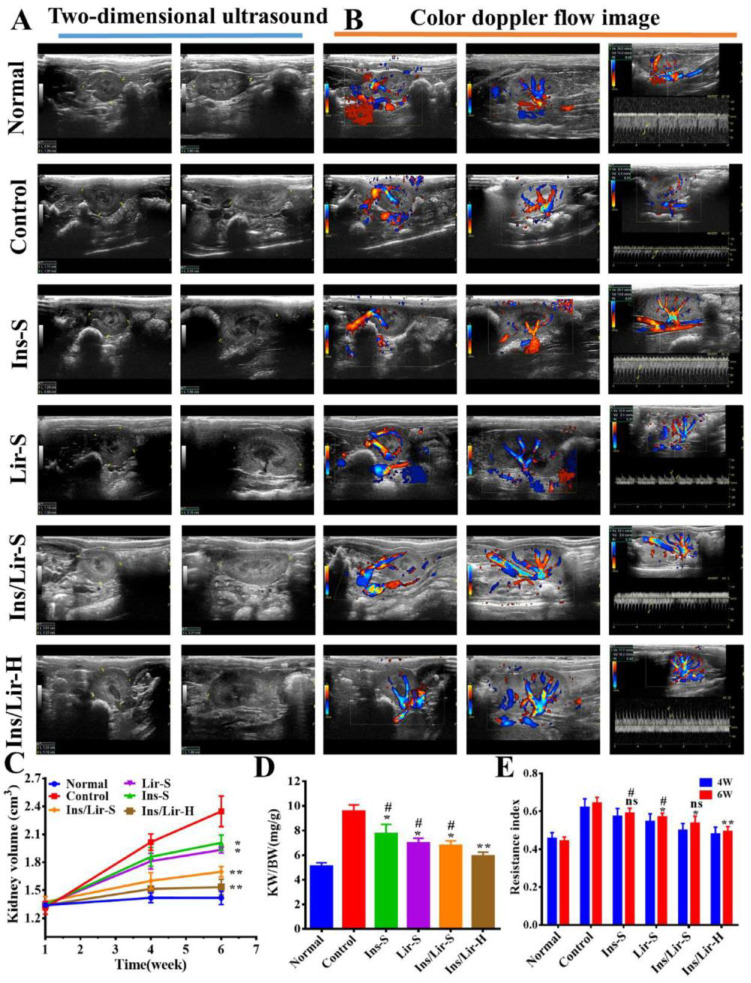
The morphological and functional recovery of the kidney of diabetic rats in the 6th week after treatment was confirmed by (**A**) 2D ultrasound imaging, (**B**) color Doppler imaging, (**C**) the calculated kidney volume based on 2D ultrasound imaging, (**D**) the calculated kidney weight/body weight ratio, and (**E**) the calculated resistance index in the 4th and 6th weeks based on color Doppler flow image (* *p* < 0.05; ** *p* < 0.01, compared to the control group; # *p* < 0.05, compared to the group treated with hydrogel incorporating insulin and liraglutide). Reprinted from [150], copyright (2021), with permission from Elsevier.

After evaluating a series of thermoresponsive hydrogels, Dutta et al. [147] selected a poly(lactide-co-glycolide)-b-poly(ethylene glycol)-b-poly(lactide-co-glycolide) hydrogel for encapsulating glucagon-like peptide (peptide A) and modified insulin analogs. When treating diabetic mice with a single SC administration of peptide A-loaded hydrogel, their blood glucose level decreased and was below 50–65% of the initial values over two to three days. For self-regulation of insulin delivery by SC injection, alginate was grafted with a temperature/glucose dual-responsive copolymer consisting of N-isopropylacrylamide and 3-acrylamidophenylboronic acid monomers, maintaining good biocompatibility [148]. It was found that insulin can be dispersed uniformly in a cold copolymer solution (10 °C), which turns into a gel in situ by raising the temperature to 37 °C. Diabetic nephropathy is a complication of type 1 and type 2 diabetes related to the progressive reduction of kidney function [149]. In a work aimed at hindering the progression of this disease, Tong et al. [150] fabricated a glucose-responsive hydrogel based on PBA-grafted γ-polyglutamic acid and konjac glucomannan to deliver insulin and liraglutide (an antidiabetic medication). In streptozotocin-induced diabetic rats receiving an SC injection of the hydrogel every three days for six weeks, morphological and functional recovery of the kidney was observed and attributed to oxidative stress reduction and autophagy activation (Figure 3).

Many apoptotic proteins, such as granzyme B (GrB), have been investigated for cancer therapy. This serine protease stored in secretory granules of activated cytotoxic T lymphocytes and natural killer cells was reported to be a highly potent mediator in the apoptotic death of cancer cells [151,152]. Therefore, Pang et al. [153] constructed a thermoresponsive hydrogel consisting of poly (ethylene glycol)-poly(γ-ethyl-L-glutamate) diblock copolymer to deliver GrB- and docetaxel-loaded mini micelles. The hydrogel was formed in situ at body temperature and gradually degraded by proteinase to release mini micelles. The ability of mini micelles to escape from lysosomes and penetrate deeply into the tumor was validated in vitro and in vivo. Further, data from studies on both SC tumor and postoperative recurrence models supported high tumor inhibition with the combination of GrB and docetaxel via peritumoral injection of the hydrogel.

**Figure 4:**
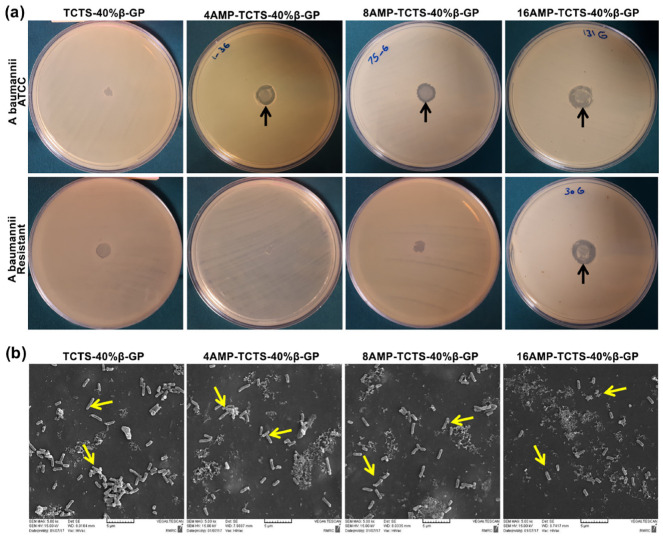
Antibacterial activity of thermoresponsive chitosan hydrogels (TCTS-40%β-GP) loaded with different concentrations of antimicrobial peptide (AMP) (0, 4, 8 and 16 μg/mL) against ATCC and resistant A. baumannii: (**a**) disk diffusion assay and (**b**) scanning electron microscope (SEM) micrographs of resistant A. baumannii bacteria grown on these hydrogels. Reprinted from [156], copyright (2020), with permission from Elsevier.

Antimicrobial peptides (AMPs) are essential components of the innate immune defense in multicellular organisms and are currently under development as novel anti-infective drugs [154]. While most AMPs kill microbial pathogens directly, others act indirectly by regulating the host’s defensive system [155]. Since an ideal skin wound dressing should have antibacterial activity against antibiotic-resistant bacteria, Rezaei et al. [156] prepared thermoresponsive chitosan hydrogels loaded with different concentrations of AMPs (4, 8 and 16 μg/mL). All hydrogels showed good compatibility with human fibroblasts. Although they had strong antibacterial activity against standard strains of Acinetobacter baumannii, only the addition of AMPs at a concentration of 16 μg/mL provided the hydrogel with effective antibacterial activity against resistant strains with no sign of cytotoxicity for human cells (Figure 4).

Other examples of stimuli-responsive hydrogels for protein delivery are described in Table 4.

## 5. Conclusions and Future Perspectives

In recent years, a variety of stimuli-responsive hydrogels have been developed for the delivery of peptides and proteins. Compared to conventional hydrogels, stimuli-responsive hydrogels provide more precise control of the location and/or duration of protein release. Considering the stimulus to which the hydrogel responds, more benefits can be added. For instance, thermoresponsive polymers allow for developing in situ gelling systems, which combine the ease of injecting low viscosity dispersions with the rapid formation of implants in situ after gelation at body temperature. An in situ-forming implant not only adapts its shape to the geometry of the injection site but also acts as a reservoir system, prolonging protein release over longer periods than preformed hydrogels. Also, the incorporation of pH-responsive polymers or enzyme-cleavable moieties can improve or confer biodegradability to the hydrogel network and enable self-regulated release that is convenient for certain diseases. However, despite their promising outcomes in vitro and in vivo, some obstacles to the clinical translation of these therapeutic systems remain.

When designing a hydrogel for drug delivery, polymer selection warrants special attention. Once the polymer system has released its payload, it should be removed from the body, which usually occurs by renal filtration. However, given its molecular weight cut-off of 40 kDa [162], polymers should be small enough to be filtered in the kidney, otherwise therapeutic applicability will depend on their long-term toxicity and immunogenicity. Besides, as with other material surfaces, nonspecific interaction with biological molecules takes place as soon as the hydrogel encounters complex fluids. Antifouling coating with polymer brushes could be a strategy to prevent nonspecific adhesion onto the hydrogel network [163].

In the case of stimuli-responsive hydrogels, researchers face even more hurdles to access materials that produce a sensitive and complete response in vivo. In contrast to exogenous stimuli, which offer precise regulation of the hydrogel’s performance at the target site, endogenous stimuli may be inconsistent in their responsive behavior. To illustrate this, the developed hydrogels might respond to the disease-associated hallmarks, but rarely are these internal cues exclusive to a single diseased site, thus leading to suboptimal selectivity in the overly complex in vivo environment. Even if they do, this shortcoming persists as biological parameters vary between individuals and over time.

To improve site-specificity and achieve fine control of peptide and protein release, future works will certainly follow the trend of fabricating hydrogels with multiple stimuli-responsiveness, which brings other challenges, such as complex polymer engineering and difficult scaling-up of production, into the equation. The road ahead seems long but full of promise as long as more experts in biology, chemistry, and medicine step in and establish effective interdisciplinary collaborations.

## Figures and Tables

**Table 1:** Some examples of protein-based parenteral dosage forms approved by the FDA.

Year	Active Ingredient Trade Name	Description	Pharmaceutical Dosage Form	Indication
2017	Etelcalcetide Parsabiv^®^	Calcium-sensing receptor agonist	Injectable solution (IV)	Hyperparathyroidism
2017	Semaglutide Ozempic^®^	Glucagon-like peptide 1 (GLP-1) receptor agonist	Injectable solution (SC)	Diabetes
2018	Tildrakizumab Ilumya^®^	Interleukin-23 antagonist	Injectable solution (SC)	Moderate-to-severe plaque psoriasis
2018	Cemiplimab Libtayo^®^	Programmed death receptor-1 (PD-1) blocking antibody	Injectable solution (IV)	Cutaneous squamous-cell carcinoma
2018	Calaspargase pegol Asparlas^®^	Asparagine-specific enzyme	Injectable solution (IV)	Acute lymphoblastic leukemia
2019	Crizanlizumab-tmca Adakveo^®^	Selectin-blocking antibody	Injectable solution (IV)	Pain caused by sickle cell disease
2020	Setmelanotide Imcivree^TM^	Melanocortin 4 (MC4) receptor agonist	Injectable solution (SC)	Chronic weight management
2020	Somapacitan-beco Sogroya^®^	Human growth hormone analog	Injectable solution (SC)	Growth hormone deficiency
2020	Ansuvimab-zykl Ebanga^TM^	Zaire ebolavirus glycoprotein (EBOV GP)-directed human monoclonal antibody	Injectable solution (IV)	Infection caused by Zaire ebolavirus
2021	Dasiglucagon Zegalogue^®^	Anti-hypoglycemic agent	Injectable solution (SC)	Severe hypoglycemia
2021	Dostarlimab-gxly Jemperli^®^	Programmed death receptor-1 (PD-1) blocking antibody	Injectable solution (IV)	Endometrial cancer
2022	Olipudase alfa Xenpozyme^TM^	Sphingomyelin-specific enzyme	Injectable solution (IV)	Acid sphingomyelinase deficiency
2023	Pegunigalsidase alfa-iwxj Elfabrio^®^	Glycosphingolipid-specific enzyme	Injectable solution (IV)	Fabry disease
2023	Somatrogon-ghla Ngenla^TM^	Human growth hormone analog	Injectable solution (SC)	Growth hormone deficiency
2023	Pozelimab-bbfg Veopoz^TM^	Recombinant IgG4 monoclonal antibody	Injectable solution (IV or SC)	CHAPLE disease

CHAPLE: CD55 deficiency with hyperactivation of complement, angiopathic thrombosis, and protein-losing enteropathy; IV: intravenous; SC: subcutaneous.

**Table 2:** Commercially available therapeutic proteins and peptides organized by route of administration.

Route	Protein or Peptide	Trade Name	Company	Indication	Ref.
Oral	Cyclosporin A	Neoral^®^	Novartis (Switzerland)	Systemic immunosuppressive therapy	[50,53]
	Pancrelipase	Creon^®^	AbbVie (USA)	Exocrine pancreatic insufficiency	
	Linaclotide	Linzess^®^	Actavis (USA)	Irritable bowel syndrome and chronic idiopathic constipation	
	Tilactase	Lacteeze^®^	Lacteeze (USA)	Lactose intolerance	
	Vancomycin	Vancocin^®^	ANI Pharmaceuticals (USA)	Infection	
	Octreotide	Mycapssa^®^	Chiasma (USA)	Long-term maintenance treatment in acromegaly patients	
	Semaglutide	Rybelsus^®^	Novo Nordisk (Denmark)	Type 2 diabetes mellitus	
Nasal	Desmopressin	DDAVP^®^	Ferring Pharmaceuticals (Switzerland)	Antidiuretic replacement therapy in the management of central diabetes insipidus	[1,56]
	Calcitonin	Miacalcin^®^	Novartis (Switzerland)	Postmenopausal osteoporosis	
		Fortical^®^	Upsher-Smith (USA)	Hypercalcemia, osteoporosis	
	Oxytocin	Syntocinon^®^	Novartis (Switzerland)	Induction of labor	
	Nafarelin	Synarel^®^	Pfizer (USA)	Central precocious puberty	
	Buserelin	Suprecur^®^	Sanofi-Aventis (FR)	Prostate cancer, endometriosis	
Pulmonary	Dornase alfa	Pulmozyme^®^	Genentech (USA)	Cystic fibrosis	[56,73]
	Insulin	Afrezza^®^	MannKind (USA)	Diabetes mellitus	
Ocular	Ranibizumab	Lucentis^®^	Genentech (USA)	Neovascular age-related macular degeneration; diabetic retinopathy	[74,75]
	Pegaptanib sodium	Macugen^®^	Eyetech Pharmaceuticals and Pfizer (USA)	Neovascular age-related macular degeneration	
	Aflibercept	Eylea^®^	Regeneron Pharmaceuticals (USA)	Neovascular age-related macular degeneration; diabetic retinopathy	
	Cenegermin	Oxervate™	Dompé (IT)	Neurotrophic keratitis treatment	
Transdermal	Insulin	Solo™	Medingo (USA)	Diabetes mellitus	[76]

**Table 3:** List of stimuli-responsive polymers organized by the stimulus.

Stimuli	Polymers	Origin/Synthesis	Ref.
Temperature	Poloxamers	Sequential polymerization of propylene oxide and ethylene oxide in the presence of alkaline catalysts	[119]
	Methyl cellulose	Reaction of alkali cellulose with methylene chloride	[120]
	Hydroxypropylcellulose	Reaction of alkali cellulose with propylene oxide	[121]
	Xyloglucan	Extraction from the seed of the tamarind tree (Tamarindus indica)	[122]
	Hydroxypropylmethylcellulose	Reaction of alkali cellulose with methylene chloride and propylene oxide	[123]
	Poly(N-isopropylacrylamide)	Free-radical polymerization of N-isopropylacrylamide	[124]
pH	Carbomers	Crosslinking of polyacrylic acids with the allyl ethers of pentaerythritol or sucrose	[125]
	Chitosan	Partial N-deacetylation of chitin	[126]
	Cellulose acetate phthalate	Reaction of a partially substituted cellulose acetate with phthalic anhydride in the presence of an organic solvent and a basic catalyst	[127]
	Sodium carboxymethyl cellulose	Reaction of alkali cellulose with sodium monochloroacetate	[120]
	Poly(L-lysine)	Biosynthesis by the bacterium strain Streptomyces albulus	[128]
	Polyvinyl sulfonic acid	Free-radical polymerization of vinyl sulfonic acid	[129]
	Polymethacrylic acid	Free-radical polymerization of methacrylic acid	[130]
Ionic strength	Gellan gum	Biosynthesis by Sphingomonas elodea	[131]
	Alginates	Extraction from brown marine algae and Pseudomonas and Azotobacter bacteria	[104]
	Xanthan gum	Biosynthesis by Xanthomonas campestris	[132]
	Carrageenan	Extraction from red seaweeds (Rhodophyta)	[133]
	Pectin	Extraction from citrus and apple fruits	[134]
	Hyaluronic acid	Biosynthesis by Streptococcus zooepidemicus and recombinant systems	[135]
Enzyme	Dextran	Biosynthesis by Leuconostoc mesenteroides NRRL B-512F	[136]
	Hyaluronic acid	Biosynthesis by Streptococcus zooepidemicus and recombinant systems	[135]
	Polyethylene glycol	Ring-opening polymerization of ethylene oxide	[137]
	Poly(allylamine)	Polymerization of allylamine	[138]

**Table 4:** Stimuli-responsive hydrogels for proteins administration.

Proteins	Stimuli Responsiveness Composition	Route	Highlights	Ref.
Vascular endothelial growth factor and monocyte chemotactic protein-1	Temperature-responsive PLGA-mPEG	Intrafemoral	Good cytocompatibility The mean vessel diameter and density increased over weeks after implantation of the HG in the necrosis site of the rabbit femoral head	[157]
Insulin	pH- and amylase-responsive CMS-g-AA/PMAA	Oral	Insulin protection in artificial gastric fluid Insulin release was accelerated in artificial intestinal fluid containing α-amylase Diabetic rats received twice-daily oral treatments for two weeks, alleviating diabetic symptoms and suppressing body weight loss	[158]
BSA and insulin	pH-responsive 4a-PEG-PLG	Oral or SC	pH-dependent release of BSA or insulin from the HG Compared to native BSA and insulin, the bioactivities of BSA and insulin released from the HG were preserved Good cytocompatibility In vivo studies showed complete degradation of the HG after eight days	[159]
Insulin	Glucose- and pH-responsive PBA, glucose oxidase and catalase	SC	When pH < pK_a_, the HG disassembled, along with insulin release In vivo studies showed biocompatibility and effectiveness in regulating blood glucose levels for a long time	[160]
FITC-BSA	Enzyme-responsive HPP-GC	SC	When lysozyme was present, degradation controlled the release of protein In vitro release studies showed minimal diffusion-controlled release and retention of the encapsulated protein within the HG	[161]

4a-PEG-PLG: 4-arm poly (ethylene glycol)-b-poly (L-glutamic acid); BSA: bovine serum albumin; CMS-g-AA: acrylate-grafted-carboxymethyl starch; FITC-BSA: fluorescein isothiocyanate-conjugated bovine serum albumin; HG: hydrogel; HPP-GC: 3-(4-hydroxyphenyl)- propionic acid-modified glycol chitosan; MAA: methacrylic acid; PBA: phenylboronic acid; PLGA-mPEG: poly (D, L-lactic-co-glycolic acid)-b-methoxy poly (ethylene glycol); SC: subcutaneous.

## References

[1] Bajracharya R., Song J.G., Back S.Y., Han H.K. (2019). Recent Advancements in Non-Invasive Formulations for Protein Drug Delivery. Comput. Struct. Biotechnol. J..

[2] Walsh G. (2018). Biopharmaceutical benchmarks 2018. Nat. Biotechnol..

[3] Asfour M.H. (2021). Advanced trends in protein and peptide drug delivery: A special emphasis on aquasomes and microneedles techniques. Drug Deliv. Transl. Res..

[4] Florence A.T., Attwood D. (2015). Physicochemical Principles of Pharmacy: In Manufacture, Formulation and Clinical Use.

[5] Lin J.H. (2009). Pharmacokinetics of biotech drugs: Peptides, proteins and monoclonal antibodies. Curr. Drug Metab..

[6] Irianti M.K., Rahmasari R., Arifianti A.E., Iswandana R. (2020). Non-invasive strategies for protein drug delivery: Oral, transdermal, and pulmonary. J. Appl. Pharm. Sci..

[7] Vermonden T., Censi R., Hennink W.E. (2012). Hydrogels for protein delivery. Chem. Rev..

[8] Bae K.H., Kurisawa M. (2016). Emerging hydrogel designs for controlled protein delivery. Biomater. Sci..

[9] Narayanaswamy R., Torchilin V.P. (2019). Hydrogels and Their Applications in Targeted Drug Delivery. Molecules.

[10] Chatterjee S., Hui P.C.-l., Lăcrămioara P., Mihaela Violeta G., Cristina-Elena D.-P. (2018). Stimuli-Responsive Hydrogels: An Interdisciplinary Overview. Hydrogels.

[11] Vasudevan D., Sreekumari S., Vaidyanathan K. (2017). Proteins: Structure and Function. Textbook of Biochemistry for Medical Students.

[12] Jain A., Jain A., Gulbake A., Shilpi S., Hurkat P., Jain S.K. (2013). Peptide and protein delivery using new drug delivery systems. Crit. Rev. Ther. Drug Carrier Syst..

[13] Deb P.K., Al-Attraqchi O., Chandrasekaran B., Paradkar A., Tekade R.K., Tekade R.K. (2019). Protein/Peptide Drug Delivery Systems: Practical Considerations in Pharmaceutical Product Development. Basic Fundamentals of Drug Delivery.

[14] de la Torre B.G., Albericio F. (2020). The Pharmaceutical Industry in 2019. An Analysis of FDA Drug Approvals from the Perspective of Molecules. Molecules.

[15] Mullard A. (2020). 2019 FDA drug approvals. Nat. Rev. Drug Discov..

[16] Mullard A. (2021). 2020 FDA drug approvals. Nat. Rev. Drug Discov..

[17] Martins A.C., Albericio F., de la Torre B.G. (2023). FDA Approvals of Biologics in 2022. Biomedicines.

[18] Carter P.J. (2011). Introduction to current and future protein therapeutics: A protein engineering perspective. Exp. Cell Res..

[19] de la Torre B.G., Albericio F. (2022). The Pharmaceutical Industry in 2021. An Analysis of FDA Drug Approvals from the Perspective of Molecules. Molecules.

[20] Leader B., Baca Q.J., Golan D.E. (2008). Protein therapeutics: A summary and pharmacological classification. Nat. Rev. Drug Discov..

[21] Timofeev V., Samygina V. (2023). Protein Crystallography: Achievements and Challenges. Crystals.

[22] Voet D., Voet J.G. (2010). Amino acids. Biochemistry.

[23] Liang W., Pan H.W., Vllasaliu D., Lam J.K.W. (2020). Pulmonary Delivery of Biological Drugs. Pharmaceutics.

[24] Awwad S., Angkawinitwong U. (2018). Overview of antibody drug delivery. Pharmaceutics.

[25] Datta-Mannan A. (2019). Mechanisms Influencing the Pharmacokinetics and Disposition of Monoclonal Antibodies and Peptides. Drug Metab. Dispos..

[26] Joseph M., Trinh H.M., Mitra A.K., Mitra A.K., Cholkar K., Mandal A. (2017). Peptide and Protein-Based Therapeutic Agents. Emerging Nanotechnologies for Diagnostics, Drug Delivery and Medical Devices.

[27] Goldberg M., Gomez-Orellana I. (2003). Challenges for the oral delivery of macromolecules. Nat. Rev. Drug Discov..

[28] Tiam F., Adam H., Remigius U.A., Ali Demir S. (2016). Noninvasive Strategies for Systemic Delivery of Therapeutic Proteins—Prospects and Challenges. Smart Drug Delivery System.

[29] Renukuntla J., Vadlapudi A.D., Patel A., Boddu S.H., Mitra A.K. (2013). Approaches for enhancing oral bioavailability of peptides and proteins. Int. J. Pharm..

[30] Vugmeyster Y., Xu X., Theil F.P., Khawli L.A., Leach M.W. (2012). Pharmacokinetics and toxicology of therapeutic proteins: Advances and challenges. World J. Biol. Chem..

[31] Jain D., Mahammad S.S., Singh P.P., Kodipyaka R. (2019). A review on parenteral delivery of peptides and proteins. Drug Dev. Ind. Pharm..

[32] Ibeanu N., Egbu R., Onyekuru L., Javaheri H., Khaw P.T., Williams G.R., Brocchini S., Awwad S. (2020). Injectables and Depots to Prolong Drug Action of Proteins and Peptides. Pharmaceutics.

[33] Usach I., Martinez R., Festini T., Peris J.E. (2019). Subcutaneous Injection of Drugs: Literature Review of Factors Influencing Pain Sensation at the Injection Site. Adv. Ther..

[34] Mueller C., Altenburger U., Mohl S. (2018). Challenges for the pharmaceutical technical development of protein coformulations. J. Pharm. Pharmacol..

[35] Jackisch C., Müller V., Maintz C., Hell S., Ataseven B. (2014). Subcutaneous Administration of Monoclonal Antibodies in Oncology. Geburtshilfe Frauenheilkd.

[36] Tomar D.S., Kumar S., Singh S.K., Goswami S., Li L. (2016). Molecular basis of high viscosity in concentrated antibody solutions: Strategies for high concentration drug product development. MAbs.

[37] Wang W., Chen N., Shen X., Cunningham P., Fauty S., Michel K., Wang B., Hong X., Adreani C., Nunes C.N. (2012). Lymphatic transport and catabolism of therapeutic proteins after subcutaneous administration to rats and dogs. Drug Metab. Dispos..

[38] Hamuro L., Kijanka G., Kinderman F., Kropshofer H., Bu D.X., Zepeda M., Jawa V. (2017). Perspectives on Subcutaneous Route of Administration as an Immunogenicity Risk Factor for Therapeutic Proteins. J. Pharm. Sci..

[39] Supersaxo A., Hein W.R., Steffen H. (1990). Effect of molecular weight on the lymphatic absorption of water-soluble compounds following subcutaneous administration. Pharm. Res..

[40] FDA: Novel Drug Approvals for 2017. https://www.fda.gov/drugs/new-drugs-fda-cders-new-molecular-entities-and-new-therapeutic-biological-products/novel-drug-approvals-2017.

[41] FDA: Novel Drug Approvals for 2018. https://www.fda.gov/drugs/new-drugs-fda-cders-new-molecular-entities-and-new-therapeutic-biological-products/novel-drug-approvals-2018.

[42] FDA: Novel Drug Approvals for 2019. https://www.fda.gov/drugs/new-drugs-fda-cders-new-molecular-entities-and-new-therapeutic-biological-products/novel-drug-approvals-2019.

[43] FDA: Novel Drugs Approvals for 2020. https://www.fda.gov/drugs/new-drugs-fda-cders-new-molecular-entities-and-new-therapeutic-biological-products/novel-drug-approvals-2020.

[44] FDA: Novel Drug Approvals for 2021. https://www.fda.gov/drugs/new-drugs-fda-cders-new-molecular-entities-and-new-therapeutic-biological-products/novel-drug-approvals-2021.

[45] FDA: Novel Drug Approvals for 2022. https://www.fda.gov/drugs/new-drugs-fda-cders-new-molecular-entities-and-new-therapeutic-biological-products/novel-drug-approvals-2022.

[46] FDA: Novel Drug Approvals for 2023. https://www.fda.gov/drugs/new-drugs-fda-cders-new-molecular-entities-and-new-therapeutic-biological-products/novel-drug-approvals-2023.

[47] Devadasu V.R., Deb P.K., Maheshwari R., Sharma P., Tekade R.K., Tekade R.K. (2018). Physicochemical, Pharmaceutical, and Biological Considerations in GIT Absorption of Drugs. Dosage Form Design Consideration.

[48] Han Y., Gao Z., Chen L., Kang L., Huang W., Jin M., Wang Q., Bae Y.H. (2019). Multifunctional oral delivery systems for enhanced bioavailability of therapeutic peptides/proteins. Acta Pharm. Sin. B.

[49] Abuhelwa A.Y., Williams D.B., Upton R.N., Foster D.J. (2017). Food, gastrointestinal pH, and models of oral drug absorption. Eur. J. Pharm. Biopharm..

[50] Wright L., Barnes T.J., Prestidge C.A. (2020). Oral delivery of protein-based therapeutics: Gastroprotective strategies, physiological barriers and in vitro permeability prediction. Int. J. Pharm..

[51] Brown T.D., Whitehead K.A., Mitragotri S. (2020). Materials for oral delivery of proteins and peptides. Nat. Rev. Mater..

[52] Linnankoski J., Mäkelä J., Palmgren J., Mauriala T., Vedin C., Ungell A.L., Lazorova L., Artursson P., Urtti A., Yliperttula M. (2010). Paracellular porosity and pore size of the human intestinal epithelium in tissue and cell culture models. J. Pharm. Sci..

[53] Zhu Q., Chen Z., Paul P.K., Lu Y., Wu W., Qi J. (2021). Oral delivery of proteins and peptides: Challenges, status quo and future perspectives. Acta Pharm. Sin. B.

[54] Turner J.R. (2009). Intestinal mucosal barrier function in health and disease. Nat. Rev. Immunol..

[55] Thwala L.N., Préat V., Csaba N.S. (2017). Emerging delivery platforms for mucosal administration of biopharmaceuticals: A critical update on nasal, pulmonary and oral routes. Expert Opin. Drug Deliv..

[56] Anselmo A.C., Gokarn Y., Mitragotri S. (2019). Non-invasive delivery strategies for biologics. Nat. Rev. Drug Discov..

[57] Balcão V., Moutinho C. (2013). Proteins and Peptides: Non-Invasive Delivery. Encyclopedia of Pharmaceutical Science and Technology.

[58] Yoshida H., Usui A., Abe Y., Goda Y., Izutsu K.-I. (2020). Relationship Between Geometric and Aerodynamic Particle Size Distributions in the Formulation of Solution and Suspension Metered-Dose Inhalers. AAPS PharmSciTech.

[59] Patton J.S., Brain J.D., Davies L.A., Fiegel J., Gumbleton M., Kim K.J., Sakagami M., Vanbever R., Ehrhardt C. (2010). The particle has landed—Characterizing the fate of inhaled pharmaceuticals. J. Aerosol. Med. Pulm. Drug Deliv..

[60] Matthews A.A., Ee P.L.R., Ge R. (2020). Developing inhaled protein therapeutics for lung diseases. Mol. Biomed..

[61] Jin L., Zhou Q.T., Chan H.K., Larson I.C., Pennington M.W., Morales R.A.V., Boyd B.J., Norton R.S., Nicolazzo J.A. (2016). Pulmonary Delivery of the Kv1.3-Blocking Peptide HsTX1[R14A] for the Treatment of Autoimmune Diseases. J. Pharm. Sci..

[62] Patton J.S., Fishburn C.S., Weers J.G. (2004). The lungs as a portal of entry for systemic drug delivery. Proc. Am. Thorac. Soc..

[63] Meng T., Kulkarni V., Simmers R., Brar V., Xu Q. (2019). Therapeutic implications of nanomedicine for ocular drug delivery. Drug Discov. Today.

[64] Bachu R.D., Chowdhury P., Al-Saedi Z.H.F., Karla P.K., Boddu S.H.S. (2018). Ocular Drug Delivery Barriers-Role of Nanocarriers in the Treatment of Anterior Segment Ocular Diseases. Pharmaceutics.

[65] Patel A., Cholkar K., Agrahari V., Mitra A.K. (2013). Ocular drug delivery systems: An overview. World J. Pharmacol..

[66] Sridhar M.S. (2018). Anatomy of cornea and ocular surface. Indian J. Ophthalmol..

[67] Gorantla S., Rapalli V.K., Waghule T., Singh P.P., Dubey S.K., Saha R.N., Singhvi G. (2020). Nanocarriers for ocular drug delivery: Current status and translational opportunity. RSC Adv..

[68] Chaulagain B., Jain A., Tiwari A., Verma A., Jain S.K. (2018). Passive delivery of protein drugs through transdermal route. Artif. Cells Nanomed. Biotechnol..

[69] Katikaneni S. (2015). Transdermal delivery of biopharmaceuticals: Dream or reality?. Ther. Deliv..

[70] Naik A., Kalia Y.N., Guy R.H. (2000). Transdermal drug delivery: Overcoming the skin’s barrier function. Pharm. Sci. Technol. Today.

[71] Morales J.O., Fathe K.R., Brunaugh A., Ferrati S., Li S., Montenegro-Nicolini M., Mousavikhamene Z., McConville J.T., Prausnitz M.R., Smyth H.D.C. (2017). Challenges and Future Prospects for the Delivery of Biologics: Oral Mucosal, Pulmonary, and Transdermal Routes. AAPS J..

[72] Yang Y., Zhou R., Wang Y., Zhang Y., Yu J., Gu Z. (2023). Recent Advances in Oral and Transdermal Protein Delivery Systems. Angew. Chem. Int. Ed. Engl..

[73] Koussoroplis S.-J., Vanbever R. (2013). Peptides and proteins: Pulmonary absorption. Encyclopedia of Pharmaceutical Science and Technology.

[74] Mandal A., Pal D., Agrahari V., Trinh H.M., Joseph M., Mitra A.K. (2018). Ocular delivery of proteins and peptides: Challenges and novel formulation approaches. Adv. Drug Deliv. Rev..

[75] FDA: FDA Approves First Drug for Neurotrophic Keratitis, a Rare Eye Disease. https://www.fda.gov/news-events/press-announcements/fda-approves-first-drug-neurotrophic-keratitis-rare-eye-disease.

[76] Selam J.L. (2010). Evolution of diabetes insulin delivery devices. J. Diabetes Sci. Technol..

[77] Wells C.M., Harris M., Choi L., Murali V.P., Guerra F.D., Jennings J.A. (2019). Stimuli-Responsive Drug Release from Smart Polymers. J. Funct. Biomater..

[78] Raza F., Zafar H., Zhu Y., Ren Y., Ullah A., Khan A.U., He X., Han H., Aquib M., Boakye-Yiadom K.O. (2018). A Review on Recent Advances in Stabilizing Peptides/Proteins upon Fabrication in Hydrogels from Biodegradable Polymers. Pharmaceutics.

[79] Pérez-Luna V.H., González-Reynoso O. (2018). Encapsulation of Biological Agents in Hydrogels for Therapeutic Applications. Gels.

[80] Shymborska Y., Budkowski A., Raczkowska J., Donchak V., Melnyk Y., Vasiichuk V., Stetsyshyn Y. (2023). Switching it Up: The Promise of Stimuli-Responsive Polymer Systems in Biomedical Science. Chem. Rec..

[81] Mahlumba P., Choonara Y.E., Kumar P., du Toit L.C., Pillay V. (2016). Stimuli-Responsive Polymeric Systems for Controlled Protein and Peptide Delivery: Future Implications for Ocular Delivery. Molecules.

[82] Chatterjee S., Chi-Leung Hui P. (2019). Review of Stimuli-Responsive Polymers in Drug Delivery and Textile Application. Molecules.

[83] Jocic D., Tourrette A., Lavric P.K., Elnashar M. (2010). Biopolymer-based Stimuli-Responsive Polymeric Systems for Functional Finishing of Textiles. Biopolymers.

[84] Marques A.C., Costa P.J., Velho S., Amaral M.H. (2021). Stimuli-responsive hydrogels for intratumoral drug delivery. Drug Discov. Today.

[85] Hatakeyama H. (2017). Recent Advances in Endogenous and Exogenous Stimuli-Responsive Nanocarriers for Drug Delivery and Therapeutics. Chem. Pharm. Bull..

[86] Sarwan T., Kumar P., Choonara Y.E., Pillay V. (2020). Hybrid Thermo-Responsive Polymer Systems and Their Biomedical Applications. Front. Mater..

[87] Matanović M.R., Kristl J., Grabnar P.A. (2014). Thermoresponsive polymers: Insights into decisive hydrogel characteristics, mechanisms of gelation, and promising biomedical applications. Int. J. Pharm..

[88] Marques A.C., Costa P.C., Velho S., Amaral M.H. (2023). Injectable Poloxamer Hydrogels for Local Cancer Therapy. Gels.

[89] Chatterjee S., Hui P.C. (2021). Review of Applications and Future Prospects of Stimuli-Responsive Hydrogel Based on Thermo-Responsive Biopolymers in Drug Delivery Systems. Polymers.

[90] Honey Priya J., Rijo J., Anju A., Anoop K.R. (2014). Smart polymers for the controlled delivery of drugs—A concise overview. Acta Pharm. Sin. B.

[91] Oak M., Mandke R., Singh J. (2012). Smart polymers for peptide and protein parenteral sustained delivery. Drug Discov. Today Technol..

[92] Altomare L., Bonetti L., Campiglio C.E., De Nardo L., Draghi L., Tana F., Farè S. (2018). Biopolymer-based strategies in the design of smart medical devices and artificial organs. Int. J. Artif. Organs.

[93] Zhang J., Jiang X., Xiang W., Xu Q., Zeng H., Zhao Y., Liu M., Wang Z., Hu X., Wang Y. (2019). Bio-responsive smart polymers and biomedical applications. J. Phys. Mater..

[94] Zha L., Banik B., Alexis F. (2011). Stimulus responsive nanogels for drug delivery. Soft Matter.

[95] Andrade F., Roca-Melendres M.M., Durán-Lara E.F., Rafael D., Schwartz S. (2021). Stimuli-Responsive Hydrogels for Cancer Treatment: The Role of pH, Light, Ionic Strength and Magnetic Field. Cancers.

[96] Rizwan M., Yahya R., Hassan A., Yar M., Azzahari A.D., Selvanathan V., Sonsudin F., Abouloula C.N. (2017). pH Sensitive Hydrogels in Drug Delivery: Brief History, Properties, Swelling, and Release Mechanism, Material Selection and Applications. Polymers.

[97] Yoshida T., Lai T.C., Kwon G.S., Sako K. (2013). pH- and ion-sensitive polymers for drug delivery. Expert Opin. Drug Deliv..

[98] Lynch C.R., Kondiah P.P.D., Choonara Y.E., du Toit L.C., Ally N., Pillay V. (2020). Hydrogel Biomaterials for Application in Ocular Drug Delivery. Front. Bioeng. Biotechnol..

[99] Ferreira N.N., Ferreira L.M.B., Cardoso V.M.O., Boni F.I., Souza A.L.R., Gremião M.P.D. (2018). Recent advances in smart hydrogels for biomedical applications: From self-assembly to functional approaches. Eur. Polym. J..

[100] El-Husseiny H.M., Mady E.A., El-Dakroury W.A., Doghish A.S., Tanaka R. (2023). Stimuli-responsive hydrogels: Smart state of-the-art platforms for cardiac tissue engineering. Front. Bioeng. Biotechnol..

[101] Feng Y., Taraban M., Yu Y.B. (2012). The Effect of Ionic Strength on the Mechanical, Structural and Transport Properties of Peptide Hydrogels. Soft Matter.

[102] Rudko M., Urbaniak T., Musiał W. (2021). Recent Developments in Ion-Sensitive Systems for Pharmaceutical Applications. Polymers.

[103] Omtvedt L.A., Kristiansen K.A., Strand W.I., Aachmann F.L., Strand B.L., Zaytseva-Zotova D.S. (2021). Alginate hydrogels functionalized with β-cyclodextrin as a local paclitaxel delivery system. J. Biomed. Mater. Res. A.

[104] Zhang H., Cheng J., Ao Q. (2021). Preparation of Alginate-Based Biomaterials and Their Applications in Biomedicine. Mar. Drugs.

[105] Sharifzadeh G., Hosseinkhani H. (2017). Biomolecule-Responsive Hydrogels in Medicine. Adv. Healthc. Mater..

[106] Cao J., Yuan P., Wu B., Liu Y., Hu C. (2023). Advances in the Research and Application of Smart-Responsive Hydrogels in Disease Treatment. Gels.

[107] Zhao L., Wang L., Zhang Y., Xiao S., Bi F., Zhao J., Gai G., Ding J. (2017). Glucose Oxidase-Based Glucose-Sensitive Drug Delivery for Diabetes Treatment. Polymers.

[108] Mantha S., Pillai S., Khayambashi P., Upadhyay A., Zhang Y., Tao O., Pham H.M., Tran S.D. (2019). Smart Hydrogels in Tissue Engineering and Regenerative Medicine. Materials.

[109] Ma Q., Zhao X., Shi A., Wu J. (2021). Bioresponsive Functional Phenylboronic Acid-Based Delivery System as an Emerging Platform for Diabetic Therapy. Int. J. Nanomed..

[110] Morariu S. (2023). Advances in the Design of Phenylboronic Acid-Based Glucose-Sensitive Hydrogels. Polymers.

[111] Lim S.L., Ooi C.-W., Low L.E., Tan W.S., Chan E.-S., Ho K.L., Tey B.T. (2020). Synthesis of poly(acrylamide)-based hydrogel for bio-sensing of hepatitis B core antigen. Mater. Chem. Phys..

[112] Li X., Duan L., Kong M., Wen X., Guan F., Ma S. (2022). Applications and Mechanisms of Stimuli-Responsive Hydrogels in Traumatic Brain Injury. Gels.

[113] Qiu Y., Park K. (2001). Environment-sensitive hydrogels for drug delivery. Adv. Drug Deliv. Rev..

[114] Di Y., Wang P., Li C., Xu S., Tian Q., Wu T., Tian Y., Gao L. (2020). Design, Bioanalytical, and Biomedical Applications of Aptamer-Based Hydrogels. Front. Med..

[115] Chang D., Ma Y., Xu X., Xie J., Ju S. (2021). Stimuli-Responsive Polymeric Nanoplatforms for Cancer Therapy. Front. Bioeng. Biotechnol..

[116] Zhao Y., Ran B., Xie X., Gu W., Ye X., Liao J. (2022). Developments on the Smart Hydrogel-Based Drug Delivery System for Oral Tumor Therapy. Gels.

[117] Chandrawati R. (2016). Enzyme-responsive polymer hydrogels for therapeutic delivery. Exp. Biol. Med..

[118] Zelzer M., Todd S.J., Hirst A.R., McDonald T.O., Ulijn R.V. (2013). Enzyme responsive materials: Design strategies and future developments. Biomater. Sci..

[119] Bonacucina G., Cespi M., Mencarelli G., Giorgioni G., Palmieri G.F. (2011). Thermosensitive Self-Assembling Block Copolymers as Drug Delivery Systems. Polymers.

[120] Pita-Vilar M., Concheiro A., Alvarez-Lorenzo C., Diaz-Gomez L. (2023). Recent advances in 3D printed cellulose-based wound dressings: A review on in vitro and in vivo achievements. Carbohydr. Polym..

[121] Younes M., Aggett P., Aguilar F., Crebelli R., Dusemund B., Filipič M., Frutos M.J., Galtier P., Gundert-Remy U., EFSA Panel on Food Additives and Nutrient Sources Added to Food (ANS) (2018). Safety of low-substituted hydroxypropyl cellulose (L-HPC) to be used as a food additive in food supplements in tablet form. EFSA J..

[122] Piqué N., Gómez-Guillén M.D.C., Montero M.P. (2018). Xyloglucan, a Plant Polymer with Barrier Protective Properties over the Mucous Membranes: An Overview. Int. J. Mol. Sci..

[123] Burdock G.A. (2007). Safety assessment of hydroxypropyl methylcellulose as a food ingredient. Food Chem. Toxicol..

[124] Yang L., Fan X., Zhang J., Ju J. (2020). Preparation and Characterization of Thermoresponsive Poly(N-Isopropylacrylamide) for Cell Culture Applications. Polymers.

[125] Iglesias N., Galbis E., Romero-Azogil L., Benito E., Lucas R., García-Martín M.G., de-Paz M.V. (2020). In-Depth Study into Polymeric Materials in Low-Density Gastroretentive Formulations. Pharmaceutics.

[126] Mathaba M., Daramola M.O. (2020). Effect of Chitosan’s Degree of Deacetylation on the Performance of PES Membrane Infused with Chitosan during AMD Treatment. Membranes.

[127] Olaru N., Olaru L. (2005). Phthaloylation of cellulose acetate in acetic acid and acetone Media. Iran. Polym. J..

[128] Manouchehri S., Zarrintaj P., Saeb M.R., Ramsey J.D. (2021). Advanced Delivery Systems Based on Lysine or Lysine Polymers. Mol. Pharm..

[129] Okayasu T., Hibino T., Nishide H. (2011). Free Radical Polymerization Kinetics of Vinylsulfonic Acid and Highly Acidic Properties of its Polymer. Macromol. Chem. Phys..

[130] Holmes P.F., Bohrer M., Kohn J. (2008). Exploration of polymethacrylate structure-property correlations: Advances towards combinatorial and high-throughput methods for biomaterials discovery. Prog. Polym. Sci..

[131] Osmałek T., Froelich A., Tasarek S. (2014). Application of gellan gum in pharmacy and medicine. Int. J. Pharm..

[132] Jadav M., Pooja D., Adams D.J., Kulhari H. (2023). Advances in Xanthan Gum-Based Systems for the Delivery of Therapeutic Agents. Pharmaceutics.

[133] Torres M.D., Flórez-Fernández N., Domínguez H. (2019). Integral Utilization of Red Seaweed for Bioactive Production. Mar. Drugs.

[134] Chandel V., Biswas D., Roy S., Vaidya D., Verma A., Gupta A. (2022). Current Advancements in Pectin: Extraction, Properties and Multifunctional Applications. Foods.

[135] Liu L., Liu Y., Li J., Du G., Chen J. (2011). Microbial production of hyaluronic acid: Current state, challenges, and perspectives. Microb. Cell Fact..

[136] Yáñez-Fernández J., Herrera Ovando M.G., Patlán Ramírez L., Ramírez-Sotelo G., Guarin C.A., Castro-Rodríguez D.C. (2021). Factorial Design to Optimize Dextran Production by the Native Strain Leuconostoc mesenteroides SF3. ACS Omega.

[137] Vojkovsky T., Sullivan B., Sill K.N. (2016). Synthesis of heterobifunctional polyethylene glycols: Polymerization from functional initiators. Polymer.

[138] Jurko L., Bračič M., Hribernik S., Makuc D., Plavec J., Jerenec F., Žabkar S., Gubeljak N., Štern A., Kargl R. (2021). Succinylation of Polyallylamine: Influence on Biological Efficacy and the Formation of Electrospun Fibers. Polymers.

[139] Municoy S., Álvarez Echazú M.I., Antezana P.E., Galdopórpora J.M., Olivetti C., Mebert A.M., Foglia M.L., Tuttolomondo M.V., Alvarez G.S., Hardy J.G. (2020). Stimuli-Responsive Materials for Tissue Engineering and Drug Delivery. Int. J. Mol. Sci..

[140] Fu X., Hosta-Rigau L., Chandrawati R., Cui J. (2018). Multi-Stimuli-Responsive Polymer Particles, Films, and Hydrogels for Drug Delivery. Chem.

[141] Cheng R., Meng F., Deng C., Klok H.A., Zhong Z. (2013). Dual and multi-stimuli responsive polymeric nanoparticles for programmed site-specific drug delivery. Biomaterials.

[142] Pham S.H., Choi Y., Choi J. (2020). Stimuli-Responsive Nanomaterials for Application in Antitumor Therapy and Drug Delivery. Pharmaceutics.

[143] Lima D.S., Tenório-Neto E.T., Lima-Tenório M.K., Guilherme M.R., Scariot D.B., Nakamura C.V., Muniz E.C., Rubira A.F. (2018). pH-responsive alginate-based hydrogels for protein delivery. J. Mol. Liq..

[144] Phan V.H.G., Thambi T., Gil M.S., Lee D.S. (2017). Temperature and pH-sensitive injectable hydrogels based on poly(sulfamethazine carbonate urethane) for sustained delivery of cationic proteins. Polymer.

[145] Sarkar S., Gulati K., Mishra A., Poluri K.M. (2020). Protein nanocomposites: Special inferences to lysozyme based nanomaterials. Int. J. Biol. Macromol..

[146] Xu H.L., Xu J., Zhang S.S., Zhu Q.Y., Jin B.H., ZhuGe D.L., Shen B.X., Wu X.Q., Xiao J., Zhao Y.Z. (2017). Temperature-sensitive heparin-modified poloxamer hydrogel with affinity to KGF facilitate the morphologic and functional recovery of the injured rat uterus. Drug Deliv..

[147] Dutta K., Das R., Ling J., Monibas R.M., Carballo-Jane E., Kekec A., Feng D.D., Lin S., Mu J., Saklatvala R. (2020). In Situ Forming Injectable Thermoresponsive Hydrogels for Controlled Delivery of Biomacromolecules. ACS Omega.

[148] Hu D.-N., Ju X.-J., Pu X.-Q., Xie R., Wang W., Liu Z., Chu L.-Y. (2021). Injectable Temperature/Glucose Dual-Responsive Hydrogels for Controlled Release of Insulin. Ind. Eng. Chem. Res..

[149] Rahmanian E., Salari N., Mohammadi M., Jalali R. (2019). Evaluation of sexual dysfunction and female sexual dysfunction indicators in women with type 2 diabetes: A systematic review and meta-analysis. Diabetol. Metab. Syndr..

[150] Tong M.Q., Luo L.Z., Xue P.P., Han Y.H., Wang L.F., Zhuge D.L., Yao Q., Chen B., Zhao Y.Z., Xu H.L. (2021). Glucose-responsive hydrogel enhances the preventive effect of insulin and liraglutide on diabetic nephropathy of rats. Acta Biomater..

[151] Lu L., Zou Y., Yang W., Meng F., Deng C., Cheng R., Zhong Z. (2015). Anisamide-Decorated pH-Sensitive Degradable Chimaeric Polymersomes Mediate Potent and Targeted Protein Delivery to Lung Cancer Cells. Biomacromolecules.

[152] Rousalova I., Krepela E. (2010). Granzyme B-induced apoptosis in cancer cells and its regulation (review). Int. J. Oncol..

[153] Pang X., Liang S., Wang T., Yu S., Yang R., Hou T., Liu Y., He C., Zhang N. (2020). Engineering Thermo-pH Dual Responsive Hydrogel for Enhanced Tumor Accumulation, Penetration, and Chemo-Protein Combination Therapy. Int. J. Nanomed..

[154] Lazzaro B.P., Zasloff M., Rolff J. (2020). Antimicrobial peptides: Application informed by evolution. Science.

[155] Mahlapuu M., Håkansson J., Ringstad L., Björn C. (2016). Antimicrobial Peptides: An Emerging Category of Therapeutic Agents. Front. Cell Infect. Microbiol..

[156] Rezaei N., Hamidabadi H.G., Khosravimelal S., Zahiri M., Ahovan Z.A., Bojnordi M.N., Eftekhari B.S., Hashemi A., Ganji F., Darabi S. (2020). Antimicrobial peptides-loaded smart chitosan hydrogel: Release behavior and antibacterial potential against antibiotic resistant clinical isolates. Int. J. Biol. Macromol..

[157] Nie L., Chang P., Sun M., Huo H., Zhang C., Ji C., Wei X., Zhou Q., Guo P., Yuan H. (2018). Composite Hydrogels with the Simultaneous Release of VEGF and MCP-1 for Enhancing Angiogenesis for Bone Tissue Engineering Applications. Appl. Sci..

[158] Liu L., Zhang Y., Yu S., Yang Z., He C., Chen X. (2018). Dual Stimuli-Responsive Nanoparticle-Incorporated Hydrogels as an Oral Insulin Carrier for Intestine-Targeted Delivery and Enhanced Paracellular Permeation. ACS Biomater. Sci. Eng..

[159] Bao X., Si X., Ding X., Duan L., Xiao C. (2019). pH-responsive hydrogels based on the self-assembly of short polypeptides for controlled release of peptide and protein drugs. J. Polym. Res..

[160] Li X., Fu M., Wu J., Zhang C., Deng X., Dhinakar A., Huang W., Qian H., Ge L. (2017). pH-sensitive peptide hydrogel for glucose-responsive insulin delivery. Acta Biomater..

[161] Gohil S.V., Padmanabhan A., Kan H.M., Khanal M., Nair L.S. (2021). Degradation-Dependent Protein Release from Enzyme Sensitive Injectable Glycol Chitosan Hydrogel. Tissue Eng. Part A.

[162] Fox M.E., Szoka F.C., Fréchet J.M.J. (2009). Soluble Polymer Carriers for the Treatment of Cancer: The Importance of Molecular Architecture. Acc. Chem. Res..

[163] Nastyshyn S., Pop-Georgievski O., Stetsyshyn Y., Budkowski A., Raczkowska J., Hruby M., Lobaz V. (2022). Protein corona of SiO_2_ nanoparticles with grafted thermoresponsive copolymers: Calorimetric insights on factors affecting entropy vs. enthalpy-driven associations. Appl. Surf. Sci..

